# Supplementary material for: HIVprotI: an integrated web based platform for prediction and design of HIV proteins inhibitors
Source: J Cheminform. 2018 Mar 9;10:12. doi: 10.1186/s13321-018-0266-y (PMC5845081; doi:10.1186/s13321-018-0266-y)
Supplement: Supplementary file 2 — Additional file 2. Source code of HIVProtI web server. [file 13321_2018_266_MOESM2_ESM.zip › HIVprotI_Source-code/hivprotI/weka-3-6-6/doc/weka/gui/arffviewer/ArffViewer.html]

ArffViewer


---


|  |  |  |
| --- | --- | --- |
| **Overview** \| **Package** \| **Class** \| **Tree** \| **Deprecated** \| **Index** \| **Help** \| **Weka's home** | |  |
| **PREV CLASS**   **NEXT CLASS** | **FRAMES**    **NO FRAMES**     **All Classes** |  |
| SUMMARY: NESTED \| FIELD \| CONSTR \| METHOD | DETAIL: FIELD \| CONSTR \| METHOD |  |


---


## weka.gui.arffviewer Class ArffViewer

```
java.lang.Object
  java.awt.Component
      java.awt.Container
          java.awt.Window
              java.awt.Frame
                  javax.swing.JFrame
                      weka.gui.arffviewer.ArffViewer
```

**All Implemented Interfaces:**: java.awt.event.WindowListener, java.awt.image.ImageObserver, java.awt.MenuContainer, java.io.Serializable, java.util.EventListener, javax.accessibility.Accessible, javax.swing.RootPaneContainer, javax.swing.WindowConstants

---

``` public class ArffViewer extends javax.swing.JFrame implements java.awt.event.WindowListener ```

A little tool for viewing ARFF files.

**Version:**
:   $Revision: 7059 $

**Author:**
:   FracPete (fracpete at waikato dot ac dot nz)

**See Also:**: Serialized Form

---

**Nested Class Summary**

**Nested classes/interfaces inherited from class java.awt.Component**

`java.awt.Component.BaselineResizeBehavior`


**Field Summary**

**Fields inherited from class javax.swing.JFrame**

`EXIT_ON_CLOSE`

**Fields inherited from class java.awt.Frame**

`CROSSHAIR_CURSOR, DEFAULT_CURSOR, E_RESIZE_CURSOR, HAND_CURSOR, ICONIFIED, MAXIMIZED_BOTH, MAXIMIZED_HORIZ, MAXIMIZED_VERT, MOVE_CURSOR, N_RESIZE_CURSOR, NE_RESIZE_CURSOR, NORMAL, NW_RESIZE_CURSOR, S_RESIZE_CURSOR, SE_RESIZE_CURSOR, SW_RESIZE_CURSOR, TEXT_CURSOR, W_RESIZE_CURSOR, WAIT_CURSOR`

**Fields inherited from class java.awt.Component**

`BOTTOM_ALIGNMENT, CENTER_ALIGNMENT, LEFT_ALIGNMENT, RIGHT_ALIGNMENT, TOP_ALIGNMENT`

**Fields inherited from interface javax.swing.WindowConstants**

`DISPOSE_ON_CLOSE, DO_NOTHING_ON_CLOSE, HIDE_ON_CLOSE`

**Fields inherited from interface java.awt.image.ImageObserver**

`ABORT, ALLBITS, ERROR, FRAMEBITS, HEIGHT, PROPERTIES, SOMEBITS, WIDTH`


**Constructor Summary**

`ArffViewer()`
  
          initializes the object


| **Method Summary** | |
| --- | --- |
| `boolean` | `getConfirmExit()`             returns the setting of whether to display a confirm messagebox or not on exit |
| `boolean` | `getExitOnClose()`             returns TRUE if a System.exit(0) is done on a close |
| `ArffViewerMainPanel` | `getMainPanel()`             returns the main panel |
| `static void` | `main(java.lang.String[] args)`             shows the frame and it tries to load all the arff files that were provided as arguments. |
| `void` | `refresh()`             validates and repaints the frame |
| `void` | `setCenteredLocation()`             positions the window at the center of the screen |
| `void` | `setConfirmExit(boolean confirm)`             whether to present a MessageBox on Exit or not |
| `void` | `setExitOnClose(boolean value)`             whether to do a System.exit(0) on close |
| `java.lang.String` | `toString()`             returns only the classname |
| `void` | `windowActivated(java.awt.event.WindowEvent e)`             invoked when a window is activated |
| `void` | `windowClosed(java.awt.event.WindowEvent e)`             invoked when a window is closed |
| `void` | `windowClosing(java.awt.event.WindowEvent e)`             invoked when a window is in the process of closing |
| `void` | `windowDeactivated(java.awt.event.WindowEvent e)`             invoked when a window is deactivated |
| `void` | `windowDeiconified(java.awt.event.WindowEvent e)`             invoked when a window is deiconified |
| `void` | `windowIconified(java.awt.event.WindowEvent e)`             invoked when a window is iconified |
| `void` | `windowOpened(java.awt.event.WindowEvent e)`             invoked when a window is has been opened |

**Methods inherited from class javax.swing.JFrame**

`getAccessibleContext, getContentPane, getDefaultCloseOperation, getGlassPane, getGraphics, getJMenuBar, getLayeredPane, getRootPane, getTransferHandler, isDefaultLookAndFeelDecorated, remove, repaint, setContentPane, setDefaultCloseOperation, setDefaultLookAndFeelDecorated, setGlassPane, setIconImage, setJMenuBar, setLayeredPane, setLayout, setTransferHandler, update`

**Methods inherited from class java.awt.Frame**

`addNotify, getCursorType, getExtendedState, getFrames, getIconImage, getMaximizedBounds, getMenuBar, getState, getTitle, isResizable, isUndecorated, remove, removeNotify, setCursor, setExtendedState, setMaximizedBounds, setMenuBar, setResizable, setState, setTitle, setUndecorated`

**Methods inherited from class java.awt.Window**

`addPropertyChangeListener, addPropertyChangeListener, addWindowFocusListener, addWindowListener, addWindowStateListener, applyResourceBundle, applyResourceBundle, createBufferStrategy, createBufferStrategy, dispose, getBufferStrategy, getFocusableWindowState, getFocusCycleRootAncestor, getFocusOwner, getFocusTraversalKeys, getGraphicsConfiguration, getIconImages, getInputContext, getListeners, getLocale, getModalExclusionType, getMostRecentFocusOwner, getOwnedWindows, getOwner, getOwnerlessWindows, getToolkit, getWarningString, getWindowFocusListeners, getWindowListeners, getWindows, getWindowStateListeners, hide, isActive, isAlwaysOnTop, isAlwaysOnTopSupported, isFocusableWindow, isFocusCycleRoot, isFocused, isLocationByPlatform, isShowing, pack, paint, postEvent, removeWindowFocusListener, removeWindowListener, removeWindowStateListener, reshape, setAlwaysOnTop, setBounds, setBounds, setCursor, setFocusableWindowState, setFocusCycleRoot, setIconImages, setLocationByPlatform, setLocationRelativeTo, setMinimumSize, setModalExclusionType, setSize, setSize, setVisible, show, toBack, toFront`

**Methods inherited from class java.awt.Container**

`add, add, add, add, add, addContainerListener, applyComponentOrientation, areFocusTraversalKeysSet, countComponents, deliverEvent, doLayout, findComponentAt, findComponentAt, getAlignmentX, getAlignmentY, getComponent, getComponentAt, getComponentAt, getComponentCount, getComponents, getComponentZOrder, getContainerListeners, getFocusTraversalPolicy, getInsets, getLayout, getMaximumSize, getMinimumSize, getMousePosition, getPreferredSize, insets, invalidate, isAncestorOf, isFocusCycleRoot, isFocusTraversalPolicyProvider, isFocusTraversalPolicySet, layout, list, list, locate, minimumSize, paintComponents, preferredSize, print, printComponents, remove, removeAll, removeContainerListener, setComponentZOrder, setFocusTraversalKeys, setFocusTraversalPolicy, setFocusTraversalPolicyProvider, setFont, transferFocusBackward, transferFocusDownCycle, validate`

**Methods inherited from class java.awt.Component**

`action, add, addComponentListener, addFocusListener, addHierarchyBoundsListener, addHierarchyListener, addInputMethodListener, addKeyListener, addMouseListener, addMouseMotionListener, addMouseWheelListener, bounds, checkImage, checkImage, contains, contains, createImage, createImage, createVolatileImage, createVolatileImage, disable, dispatchEvent, enable, enable, enableInputMethods, firePropertyChange, firePropertyChange, firePropertyChange, firePropertyChange, firePropertyChange, firePropertyChange, getBackground, getBaseline, getBaselineResizeBehavior, getBounds, getBounds, getColorModel, getComponentListeners, getComponentOrientation, getCursor, getDropTarget, getFocusListeners, getFocusTraversalKeysEnabled, getFont, getFontMetrics, getForeground, getHeight, getHierarchyBoundsListeners, getHierarchyListeners, getIgnoreRepaint, getInputMethodListeners, getInputMethodRequests, getKeyListeners, getLocation, getLocation, getLocationOnScreen, getMouseListeners, getMouseMotionListeners, getMousePosition, getMouseWheelListeners, getName, getParent, getPeer, getPropertyChangeListeners, getPropertyChangeListeners, getSize, getSize, getTreeLock, getWidth, getX, getY, gotFocus, handleEvent, hasFocus, imageUpdate, inside, isBackgroundSet, isCursorSet, isDisplayable, isDoubleBuffered, isEnabled, isFocusable, isFocusOwner, isFocusTraversable, isFontSet, isForegroundSet, isLightweight, isMaximumSizeSet, isMinimumSizeSet, isOpaque, isPreferredSizeSet, isValid, isVisible, keyDown, keyUp, list, list, list, location, lostFocus, mouseDown, mouseDrag, mouseEnter, mouseExit, mouseMove, mouseUp, move, nextFocus, paintAll, prepareImage, prepareImage, printAll, removeComponentListener, removeFocusListener, removeHierarchyBoundsListener, removeHierarchyListener, removeInputMethodListener, removeKeyListener, removeMouseListener, removeMouseMotionListener, removeMouseWheelListener, removePropertyChangeListener, removePropertyChangeListener, repaint, repaint, repaint, requestFocus, requestFocusInWindow, resize, resize, setBackground, setComponentOrientation, setDropTarget, setEnabled, setFocusable, setFocusTraversalKeysEnabled, setForeground, setIgnoreRepaint, setLocale, setLocation, setLocation, setMaximumSize, setName, setPreferredSize, show, size, transferFocus, transferFocusUpCycle`

**Methods inherited from class java.lang.Object**

`equals, getClass, hashCode, notify, notifyAll, wait, wait, wait`

**Methods inherited from interface java.awt.MenuContainer**

`getFont, postEvent`

**Constructor Detail**

### ArffViewer

```
public ArffViewer()
```

:   initializes the object


**Method Detail**

### setCenteredLocation

```
public void setCenteredLocation()
```

:   positions the window at the center of the screen

---


### setConfirmExit

```
public void setConfirmExit(boolean confirm)
```

:   whether to present a MessageBox on Exit or not

    :   **Parameters:**: `confirm` - whether a MessageBox pops up or not to confirm exit

---


### getConfirmExit

```
public boolean getConfirmExit()
```

:   returns the setting of whether to display a confirm messagebox or not
    on exit

    :   **Returns:**: whether a messagebox is displayed or not

---


### setExitOnClose

```
public void setExitOnClose(boolean value)
```

:   whether to do a System.exit(0) on close

    :   **Parameters:**: `value` - enables/disables the System.exit(0)

---


### getExitOnClose

```
public boolean getExitOnClose()
```

:   returns TRUE if a System.exit(0) is done on a close

    :   **Returns:**: true if System.exit(0) is done

---


### getMainPanel

```
public ArffViewerMainPanel getMainPanel()
```

:   returns the main panel

    :   **Returns:**: the main panel

---


### refresh

```
public void refresh()
```

:   validates and repaints the frame

---


### windowActivated

```
public void windowActivated(java.awt.event.WindowEvent e)
```

:   invoked when a window is activated

    :   **Specified by:**: `windowActivated` in interface `java.awt.event.WindowListener`
    :   **Parameters:**: `e` - the window event

---


### windowClosed

```
public void windowClosed(java.awt.event.WindowEvent e)
```

:   invoked when a window is closed

    :   **Specified by:**: `windowClosed` in interface `java.awt.event.WindowListener`
    :   **Parameters:**: `e` - the window event

---


### windowClosing

```
public void windowClosing(java.awt.event.WindowEvent e)
```

:   invoked when a window is in the process of closing

    :   **Specified by:**: `windowClosing` in interface `java.awt.event.WindowListener`
    :   **Parameters:**: `e` - the window event

---


### windowDeactivated

```
public void windowDeactivated(java.awt.event.WindowEvent e)
```

:   invoked when a window is deactivated

    :   **Specified by:**: `windowDeactivated` in interface `java.awt.event.WindowListener`
    :   **Parameters:**: `e` - the window event

---


### windowDeiconified

```
public void windowDeiconified(java.awt.event.WindowEvent e)
```

:   invoked when a window is deiconified

    :   **Specified by:**: `windowDeiconified` in interface `java.awt.event.WindowListener`
    :   **Parameters:**: `e` - the window event

---


### windowIconified

```
public void windowIconified(java.awt.event.WindowEvent e)
```

:   invoked when a window is iconified

    :   **Specified by:**: `windowIconified` in interface `java.awt.event.WindowListener`
    :   **Parameters:**: `e` - the window event

---


### windowOpened

```
public void windowOpened(java.awt.event.WindowEvent e)
```

:   invoked when a window is has been opened

    :   **Specified by:**: `windowOpened` in interface `java.awt.event.WindowListener`
    :   **Parameters:**: `e` - the window event

---


### toString

```
public java.lang.String toString()
```

:   returns only the classname

    :   **Overrides:**: `toString` in class `java.awt.Component`
    :   **Returns:**: the classname

---


### main

```
public static void main(java.lang.String[] args)
                 throws java.lang.Exception
```

:   shows the frame and it tries to load all the arff files that were
    provided as arguments.

    :   **Parameters:**: `args` - the commandline parameters **Throws:**: `java.lang.Exception` - if something goes wrong


---


|  |  |  |
| --- | --- | --- |
| **Overview** \| **Package** \| **Class** \| **Tree** \| **Deprecated** \| **Index** \| **Help** \| **Weka's home** | |  |
| **PREV CLASS**   **NEXT CLASS** | **FRAMES**    **NO FRAMES**     **All Classes** |  |
| SUMMARY: NESTED \| FIELD \| CONSTR \| METHOD | DETAIL: FIELD \| CONSTR \| METHOD |  |


---
